# Different Ways to Apply a Measurement Instrument of E-Nose Type to Evaluate Ambient Air Quality with Respect to Odour Nuisance in a Vicinity of Municipal Processing Plants

**DOI:** 10.3390/s17112671

**Published:** 2017-11-19

**Authors:** Bartosz Szulczyński, Tomasz Wasilewski, Wojciech Wojnowski, Tomasz Majchrzak, Tomasz Dymerski, Jacek Namieśnik, Jacek Gębicki

**Affiliations:** 1 Department of Chemical and Process Engineering, Chemical Faculty, Gdansk University of Technology, 11/12 G. Narutowicza Str., 80-233 Gdańsk, Poland; bartosz.szulczynski@pg.gda.pl; 2 Department of Inorganic Chemistry, Faculty of Pharmacy, Medical University of Gdańsk, Al. Hallera 107, 80-416 Gdańsk, Poland; tomwasil@gumed.edu.pl; 3 Department of Analytical Chemistry, Chemical Faculty, Gdansk University of Technology, 11/12 G. Narutowicza Str., 80-233 Gdańsk, Poland; wojciech@wojnowski.info (W.W.); tomasz.majchrzak92@gmail.com (T.M.); tomasz.dymerski@pg.gda.pl (T.D.); jacek.namiesnik@pg.edu.pl (J.N.)

**Keywords:** sensors and biosensors, bioelectronic nose, robots, drones, portable devices, odorants

## Abstract

This review paper presents different ways to apply a measurement instrument of e-nose type to evaluate ambient air with respect to detection of the odorants characterized by unpleasant odour in a vicinity of municipal processing plants. An emphasis was put on the following applications of the electronic nose instruments: monitoring networks, remote controlled robots and drones as well as portable devices. Moreover, this paper presents commercially available sensors utilized in the electronic noses and characterized by the limit of quantification below 1 ppm *v*/*v*, which is close to the odour threshold of some odorants. Additionally, information about bioelectronic noses being a possible alternative to electronic noses and their principle of operation and application potential in the field of air evaluation with respect to detection of the odorants characterized by unpleasant odour was provided.

## 1. Introduction

**Figure 1:**
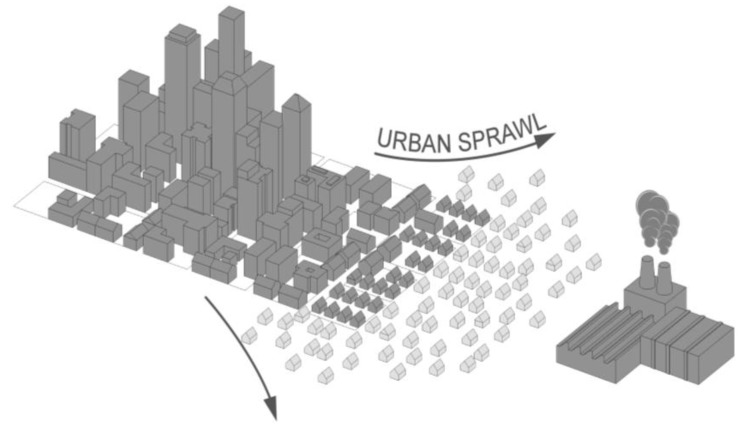
Schematic presentation of interaction between urban agglomeration and municipal processing plants.

Odorants are the chemical substances which stimulate the olfactory system of humans, so an odour is sensed. Some odorants are characterized by an unpleasant odour and their persistent or periodical presence in ambient air results in an odour nuisance. Such situations are classified as one of the main reasons for public complaints filed to various types of institutions [1,2,3]. The complexity of the problem of odour nuisance in ambient air was emphasized in a number of different reports and classified as one of the most important issues regarding environmental pollution. The human nose is a natural sensor for the detection of odorants characterized by unpleasant odour. Information about odour can be perceived by humans in a conscious and subconscious way. The odorants characterized by unpleasant odour are naturally associated with potential danger, feeling of discomfort and can cause negative psychosomatic symptoms. Thus, atmospheric emission of the gases polluted with malodorous substances constitutes a significant environmental problem [4,5,6,7,8]. Unfortunately, continuous economic and industrial progress of many countries significantly contributes to the increased emission of pollutants into the atmosphere. Expansion of urban agglomerations in regions where municipal processing plants, such as sewage treatment plants or municipal landfills, are located see increased odour problems [9,10,11,12,13]. These plants generate a substantial amount of substances that are harmful to the environment, characterized by various physical as well as chemical properties; some of these pollutants are odorants characterized by unpleasant odour. This situation still occurs despite the fact that many countries intensively implement modern technologies aimed at limiting the malodorous substances emitted from that type of plant. The negative influence of these compounds on the environment can result from: properties of particular chemical compounds, mutual interactions between the components of gas mixtures (the phenomenon of synergism) and atmospheric factors including air temperature, wind speed and direction, air humidity, insolation [14,15]. Figure 1 provides schematic information about the expansion of urban agglomerations and the related permanent or temporary odour nuisance over a particular area.

**Figure 2:**
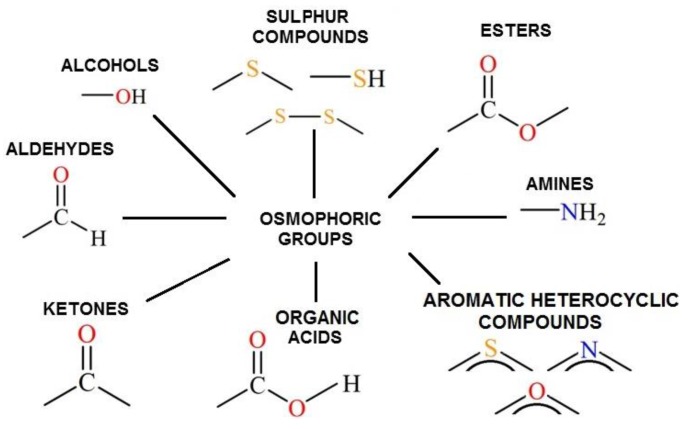
Exemplary osmophore groups of the chemical compounds which occur in ambient air in a vicinity of sewage treatment plants and municipal landfills.

The content of suitable osmophore groups in the chemical structure of odorants determines their sensory perception with respect to odour sort (what the odour of the particular odorant resembles) as well as hedonic tone (subjective opinion about the odour expressed using pleasant/unpleasant categories). Odour threshold is also a parameter describing properties of odorants. It is the minimum concentration of the odorant which stimulates the olfactory system of humans. Figure 2 presents exemplary osmophore groups of the chemical compounds that are the most frequent in the ambient air in the vicinity of municipal plants. Table 1 gathers the information about the concentrations at which odour thresholds occur [16,17,18,19,20,21,22,23,24,25].

Currently, reduction of the emission of odorants characterized by unpleasant odour is a priority for these branches of industry which introduce odorous compounds to the atmosphere. The most effective methods of odorants characterized by unpleasant odour emission abatement include:

- prevention of malodorous compounds emission via application of the best available technology (BAT),
- implementation of deodorization systems in existing industrial plants,
- proper planning at the localization and construction stage of newly-built plants.

However, independently of the applied technology of odorants characterized by unpleasant odour emission limitation, it is necessary to provide verification via monitoring of deodorization effectiveness. Evaluation of the deodorization level can be performed via measurement of odour intensity or odour concentration at an inlet or an outlet of a given technological installation. Instrumental techniques, apart from the olfactometric techniques—dynamic olfactometry, in particular, is one of the most popular methods of odour measurement. Electronic nose technique belongs to the group of instrumental methods. Similarly to the olfactometric techniques, it utilizes holistic analysis, without a need for identification of particular components contributing to a summary odour of the mixture. This technique belongs to dynamically developing instrumental techniques and it is increasingly applied for monitoring and evaluation of the effectiveness of deodorization of unpleasant odours generated by different fields of human activity [29,30,31,32,33,34,35,36].

**Figure 3:**
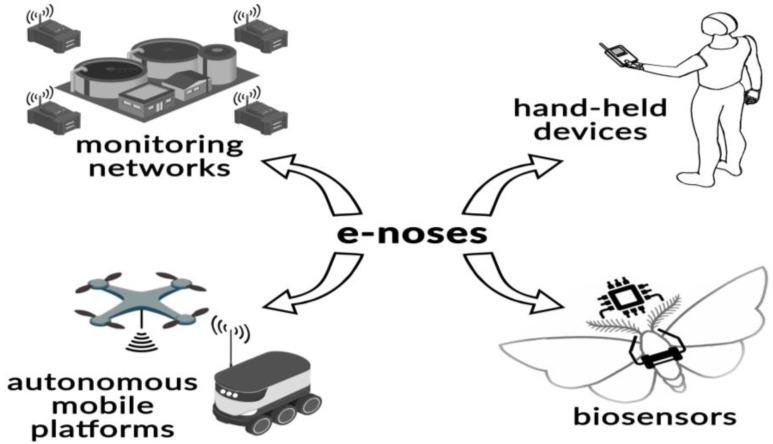
Schematic presentation of main applications of electronic nose instruments for evaluation of ambient air quality in a vicinity of municipal processing plants.

In this review paper, the authors would like to focus on four aspects connected with monitoring and evaluation of air odour quality in a vicinity of municipal processing plants using the electronic nose technique. Figure 3 schematically illustrates the ways in which an electronic nose is used for evaluation of ambient air quality. The first group includes stationary monitoring with a network of electronic nose instruments. Information from particular electronic nose units located in a given area is transferred to a central unit. The second group of application is remote robots and drones which are sent to the places that are inaccessible, dangerous or harmful to people. Another group are portable electronic nose instruments which are devices for localization of emission sources or leakages in technological installations. The fourth and final group are bioelectronic noses which, as opposed to the classic electronic noses equipped with chemical sensors, possess biosensors or biological materials sensitive to odours. This group is a promising one that is believed to be capable of replacing animals and be characterized by better selectivity than the classic electronic noses.

## 2. Sensors and Biosensors Utilized in Electronic and Bioelectronic Nose Instruments Design

Based on the information contained in Table 1, it can be noticed that volatile organic compounds (VOCs) are a dominant group of odorous compounds. These compounds, depending on the contained osmophore group, are characterized by a defined concentration range at which it is possible to identify an odour with a probability of 0.5. The value of the olfactory threshold determines the application of a suitable chemical sensor in the electronic nose instrument in order to allow measurement at a given concentration level. A range of VOCs imission concentrations which can possibly occur in ambient air is from 0.01 ppb to the maximum of single ppm. In this case, only a small number of the chemical sensors for VOCs measurement can fulfil the requirements concerning measurement at such a low concentration level. Electrochemical sensors, solid electrolyte semiconductor sensors and PID-type sensors are dominant chemical sensors for VOCs detection which are commercially available. Table 2 presents basic information concerning commercially available sensor types and their metrological parameters as far as measurement of the gases from VOCs group characterized by the limit of detection lower than 1 ppm *v*/*v* is concerned [37,38]. 

A fundamental element in the bioelectronic noses design is olfactory receptors (ORs) or cells exhibiting expression of olfactory receptor proteins which are used as an active element of the sensor in order to analyse odours with desired sensitivity and specificity. A sensitive element made of this type of biomaterial is directly connected with a sensor for odour identification and conversion of the biological signal into an analytically useful signal—electrical or optical. The sensors utilized in this type of nose are comprised of two elements—primary and secondary transducers. The first one is built from olfactory receptors cells, for instance, whereas the secondary transducer (transductor) is a non-biological device. Due to the implementation of biological elements of olfactory systems, it is possible not only to detect odorous substances at low concentration levels [39,40] but also to predict the mechanisms of odours perception. Recording and processing of the biological signal can be accomplished using: microelectrode arrays (MEAs), electrochemical impedance spectroscopy (EIS), quartz crystal microbalance (QCM), field effect transistors (FET), surface plasmon resonance (SPR) sensors and conducting polymers (for example polypyrrole), carbon nanotubes, graphene and others [41].

## 3. Stationary Monitoring Using Electronic Nose Network

In the case of this type of application, the electronic noses are located over the area where the emission of odorants characterized by unpleasant odour occurs. An indispensable element of such a network is the application of data loggers which allow acquisition of complete measurement data from the sensors including raw data and a series of data describing measurement conditions and operating parameters of a device in order to verify the quality of results and improve device functioning. Thanks to transmission connections, the data logger provides scientific staff and operators who stay in an office with remote access to the e-nose installed in measurement stations. It allows on-line acquisition and verification of measurement results as well as supervision and control over the measurement process. Data loggers, due to their superior computational power as compared to the processors controlling measurement process, can perform necessary processing of measurement data. Data transmission is realized using Internet technologies, which provides easy and fast access to the e-nose instrument. Operation of stationary electronic nose instrument can be maintenance-free, and the device works in two modes:

- the first one—sampling of ambient air for ca. 1 min,
- the second one—e-nose sensors conditioning for ca. 5–10 min.

**Figure 4:**
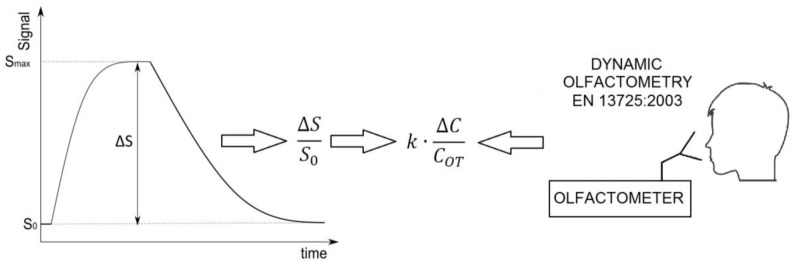
The process of sensor signal conversion into odour concentration.

In the first mode, the air sucked into a sensor chamber triggers reaction of the sensors to a change in air composition. Temperature and relative humidity of air should be stabilized before entering the sensors chamber. In the second mode, there is a flow of air stream free of odorous compounds either from a bottle or from a zero air generator, instead of ambient air purified from odorous compounds. The aim is to bring the sensor’s signals to their initial value (prior to measurement). The obtained signal is processed using converters and obtained information can be presented as odour concentration. A general overview of the process of sensor signal conversion into odour concentration is schematically presented in Figure 4. Precise determination of odour concentration from the information obtained using the sensors requires calibration with olfactometric methods. Such a situation results in the fact that a ratio of sensor signal changes ΔS to reference signal S_0_ is not directly proportional to a ratio of odour concentration ΔC to the concentration, at which olfactory threshold occurs (C_OT_). Maintenance of proportionality requires the application of correction (k), which is determined using the olfactory methods. The devices activate an alarm in case of occurence of odour nuisance or instantaneous increase in odour concentration above defined admissible value. Literature provides information about such an application of an electronic nose instrument as an element of the monitoring network in a vicinity of municipal processing plants [42,43,44]. The information about the type of electronic nose, method of measurement, and applied data analysis can be found in Table 3.

## 4. Remote Controlled Robots and Drones

The use of electronic olfaction facilitates automation of labour-intensive measurements, such as environmental odour monitoring [45]. However, when conducting measurements in an outdoor environment, an assumption that chemical substances are moved predominantly by molecular diffusion is no longer valid, as factors such as temperature, relative humidity and, most importantly, wind speed and direction come into play. For that reason, it is difficult to predict the behaviour of the plume, even with sophisticated models and ample computational power [46]. Because of that, when collecting input data for gas distribution modelling (GDM) from a distributed network of stationary electronic noses intended for environmental monitoring data from an extended period of time, fluctuations in local atmospheric conditions are taken into account. However, there are instances in which there is a need to investigate gas distribution in an area which is not covered by a pre-existing monitoring network or to rapidly detect small, localised emissions, for instance, a leak in an industrial plant. In such a case, mobile robots equipped with electronic noses can be used to map the gas distribution in a relatively short amount of time, or even be programmed to track sources of contamination. Such an application is also relevant when reducing the cost of human labour is of major concern, or in hazardous environments where they can be used over prolonged periods of time. Mobile robots can also be potentially used to collect measurements in a tighter grid than is the case with stationary e-nose networks [47]. Electronic noses could also be mounted on existing autonomous mobile devices already used in the industry, such as robots for sampling in wastewater plants [48], further reducing the operational expense.

Furthermore, in areas which are not easily accessible even for dedicated mobile platforms, aerial drones, or even swarms thereof can be used [49,50]. Such a solution would also facilitate preparing three-dimensional GDM models, and its implementation prospects are improved by the development of new, miniaturized sensor types which could be used instead of the bulky and power-consuming MOS sensors [51,52]. The turbulent flow of air usually breaks up into discrete areas of increased concentration with little continuity to the source which means that mapping of the plume is a non-trivial task, and considerable effort is put into the development of strategies for source detection, which were described in detail by Russell et al. [53]. GDM can also be approached without any strong assumptions and simplifications regarding the character of the odour plume, instead treating sensor response signals as variables in order to obtain an overview of the gas dispersion [47]. This ‘model-free’ approach was likely first used by Ishida et al. to estimate the location of emission source remotely, without reaching the source itself [54]. As is the case with the construction of electronic noses and bio-electronic olfaction, the algorithms which govern the robot’s path, either during mapping or localization are also inspired by nature. These include foraging and mate-seeking patterns exhibited by seabirds, lobsters, fish, bacteria and, perhaps most importantly, insects [55,56,57,58]. In particular, the behaviour of moths which move directly upwind, and upon losing the scent perform a local search until it is reacquired has been emulated in several approaches [59,60]. An actual olfactory apparatus of a silkworm moth was even integrated with a mobile robot for pheromone tracking [61]. However, it has been argued that biomimetic approaches to plume source tracking are not practical since the sensing capabilities of animals differ significantly from the electronic noses mounted on mobile platforms. Hernandez Bennetts et al. compared the chemical sensing available to both moths and robots and also investigated three different mobile platforms, both wheeled and aerial. They argued that the design of robots equipped with gas sensors should not directly follow the biological counterparts, but instead be guided by a thorough understanding of how the underlying principles of sensing and actuation principles of the animal olfactory sense [62]. Another issue is the fact that gas distribution modelling is usually used for mapping the concentration of a single gas, which in a real, outdoor environment is unrealistic. In actuality, there are numerous interferences which might impact the response signal of non- or semi-selective gas sensors and mask the presence of other sources of emission. A majority of reported attempts at equipping autonomous robots with gas sensors involved a single type of odour source, emitting a known chemical compound at a steady rate. In field use, in applications such as detection of sources of malodours, it might be necessary to use an array of several sensors and interpret their response pattern holistically—equipping the mobile platform with an electronic nose instead of a single sensor. This has been done in numerous studies, however in a number of these the e-nose mounted on a pre-existing robot body was used as just a set of different sensors, with only the signal magnitude of one of them used as input for GDM and source localization, with no statistical processing of the signal from the entire array, which does not conform to the definition put forward by Persaud and Dodd [63]. 

However, some attempts have been made to use an e-nose equipped robot in an environment with more than one odour type. These include several investigations involving researchers from the Center for Applied Autonomous Sensor Systems of the Örebro University, in which the capabilities of robots for odour source localization and mapping and the detection and identification of odours using an electronic nose were successfully combined [62,64,65,66]. In a recent work, a robot-mounted electronic nose equipped with an array of MOS sensors was used to generate a distribution map for multiple volatile compounds with a ‘model-free’ algorithm, together with a set of classification maps in which the estimated likelihood of detecting a given compound at a particular location is shown [47]. Such an approach could possibly find application in measuring the emission from a large, non-homogenous source of emission such as a communal landfill. A robot could also be used during landfill rehabilitation for examining the condition of the soil in a given locality. The use of electronic noses mounted on mobile autonomous devices in environmental monitoring has some promising prospective applications. A majority of research in this area is preliminary, as the challenges of using electronic olfaction in an open environment are compounded by the spatial and temporal variability of the measurements and with the necessity to develop reliable, adaptive algorithms governing the robot’s behaviour. However, with the ongoing miniaturisation of gas sensors and a steady increase of computational power at our disposal, the future use of aerial drones, likely working in swarms [59], could be used to rapidly assess the odour distribution over a relatively large area with no pre-existing monitoring network. Table 3 presents exemplary applications of robots for evaluation of air quality in a vicinity of municipal processing plants.

## 5. Portable Electronic Nose Instruments

The concept of application of portable electronic nose instruments resembles utilization of remote robots and drones. The only difference is the fact that the electronic nose is transported by humans. These devices can be employed when odour concentrations are low and do not impose a hazard to human health and life. Thus, it is rather a warning device or an instrument for detection of leakages from technological lines. Moreover, portable electronic noses can be useful in odour evaluation in the cases where other analytical methods are difficult to apply. The examples can be diffuse sources, such as sheds, hangars, hen houses, tanks etc. with stagnant air, where evaluation of emitted air stream is difficult or emission fluctuates over time, which makes attribution of odour concentration to a particular time of day impossible. Metal oxide sensors, which are the most popular and widely available chemical sensors used in electronic olfaction, are far from ideal for use in hand-held devices for environmental monitoring due to high power consumption and susceptibility to changes in relative humidity [67] but can be easily miniaturised, especially in the case of metal oxide semiconductor field effect transistors (MOSFET) [68]. The main issue with using portable electronic noses is the fact that in environmental samples, the analytes are present at very low concentrations and cannot be easily pre-concentrated, as the implementation of purge-and-trap modules would lead to excessive power consumption and bulkiness for a hand-held device. Because of that, when the atmospheric air is introduced directly into the sensor chamber, the operator must get relatively close to the odour source in order to perform a reliable measurement. It is estimated that hand-held electronic noses exhibit approximately 10 times lower precision and 10 times higher limits of detection than stationary devices [69]. On the other hand, the use of portable and hand-held devices enables on-site screening which can be followed by an in-depth analysis in the laboratory and is particularly useful in situations which require an immediate response [70]. Some studies have focused on the use of portable e-noses for detection of malodours from animal farms and landfills [71,72], however, mixed results were obtained. For the above-mentioned reasons, a vast majority of hand-held e-noses use an enclosed, static headspace sampling system and are intended for the analysis of food and agricultural products [73,74,75], and models in which dynamic sampling of atmospheric air are used are intended for analysis of localised gas leaks and emissions [76]. Exemplary applications of portable electronic nose instruments for evaluation of air odour quality are shown in Table 3.

## 6. Bioelectronic Noses

A subgroup of electronic noses engulfs bioelectronic noses which also possess high development potential. They can be implemented in all the fields where emitted odours or other types of chemical information call for high measurement sensitivity and selectivity. Intense development of these devices is aimed at elaboration of:

- sensors characterized by high sensitivity, repeatability, specificity and short response time,
- methods of detection of the wide spectrum of odorous substances comprising odour profile, including small size molecules (>300 Da),
- data processing systems enabling real-time monitoring,
- portable devices (sensor-on-chip),
- methods of production and immobilization of olfaction-inspired biomaterials on secondary transducers,
- sensor systems generating the signals similar to the ones present in biological counterparts,
- an electronic system, which would imitate human nose or brain,
- more friendly sensor systems allowing reduction of equipment and analysis costs.

**Figure 5:**
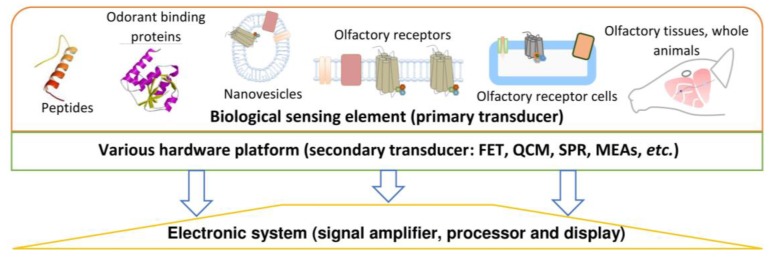
Schematic presentation of available biomaterials used for the construction of bioelectronic noses.

Regarding sensors design, one can distinguish the bioelectronic noses with different types of sensitive element plating. It was presented schematically in Figure 5.

Currently the market does not offer commercial odour biosensors based on olfactory receptors, however, there are still attempts to broaden their practical application, including in the field of environmental analytics and monitoring. Concept versions of bioelectronic noses are designed to enable detection of odorants at low concentrations before their amount reaches the level dangerous for the environment. A fundamental problem connected with proper operation of bioenoses is the poor activity of biological elements in dry conditions. A solution to this problem seems to be the implementation of synthetic peptides mimicking binding sites of the olfactory receptors [90,91]. The bioenose designed by Lee et al. [92] was capable of selective detection of trimethylamine and ammonia which are considered environmentally onerous due to their strong odour. Lee et al. also demonstrated a sensor functionalized with olfactory receptor proteins, activated in dry state [93]. Additionally, the sensor exhibited the properties of the human sense of smell such as antagonism. In order to obtain a more stable tertiary structure of the olfactory receptors, proteins were trapped in a ‘nanodisc’, a self-assembling nano-scale membrane assembly [94]. In the future, the bioelectronic nose can significantly complement the shortages of electronic noses, especially as far as more specific and sensitive analysis of numerous environmental pollutants is concerned. Son et al. [95] presented the bioelectronic nose enabling real-time monitoring of water quality via analysis of geosmin and 2-methylisoborneol as the indicators of water contamination level. In order to eliminate the disadvantages of conventional methods, human olfactory receptors (hORs) were applied, which selectively bind odorants molecules; the analysis stage is not preceded by a sample preparation process. The olfactory receptors were trapped in the form of nanovesicles deposited on single-walled carbon nanotubes (swCNT) of a field-effect transistor (FET). Another example of the biosensor based on TNT-binding receptors (tryptophan-histidine-tryptophan—WHW) linked to a conjugated polymer polydiacetylene (PDA) and modified onto the surface of single-wall carbon nanotube (SWNT)-FET, which allows measurement of 2,4,6-trimethyltoluene (TNT) content in analysed air already at the level of 1fM was presented by Kim et al. [96]. The insect antenna-based odour sensors can be a useful tool in protection plants; they reveal significant development potential in the field of identification of plant diseases markers or early detection of fires [97,98]. Highly sensitive and fast detection of volatile chemical compounds combined with substantial miniaturization potential creates big opportunities for elaboration of the odour biosensors, which can operate in the field and in turn could facilitate and broaden their practical application in environmental monitoring and analytics. Another milestone in malodorous substances analysis can be standardization of odours which is a correlation between data obtained from sensor matrix (for example bioelectronic nose) and particular sensations connected with the smell of given odorants. The legal acts concerning odour nuisance implemented in developed countries are based mainly on the investigations utilizing sensory analysis, which are still the most popular, however, they are gradually substituted with more advanced techniques, not burdened with problems associated with sensory panels [99]. Nowadays, various investigations are carried out which are aimed at digitalization of olfactory sensations and emotions accompanying particular odours and flavours [100]. Some stages can be realized using the bioelectronic noses which most precisely imitate the principle of operation of the human sense of smell thanks to the utilization of the olfactory receptors as one of the measurement elements [101].

## 7. Summary

Legislative bodies of most highly developed countries undertake the problem of the admissible level of odorous pollutants emission. The regulations concerning measurement of emission of malodours are continuously changed and improved, and electronic noses become one of the devices admitted in reference methodologies for odours measurement [102,103,104]. This paper presents different ways to apply a measurement instrument of e-nose type to evaluate ambient air with respect to detection of the odorants characterized by unpleasant odour in a vicinity of municipal processing plants. This problem is very important because the expansion of urban agglomerations goes beyond safe limits resulting in exposure to odour nuisance originating from the municipal processing plants. Due to the specificity of electronic noses, the operation of these devices can be a successful complement to current techniques of odours measurement and many countries put an emphasis on this type of approach. The electronic nose can be used as a complementary device with respect to other analytical techniques, in particular sensory analysis techniques. Currently, the market offers devices for detection of odorants characterized by unpleasant odour. Moreover, they are continuously being improved and new, upgraded prototype versions are elaborated. The most crucial aspect limiting widespread application of electronic noses is lack of defined legal regulations and their standardization. In recent years an effort has been made to develop a framework for the standardization of the use of e-noses as monitoring devices. In particular, the CEN/TC264/WG41 work group aims to propose a new European standard for instrumental odour monitoring [105]. It will be focused on technical aspects of the devices, e.g., sensitivity and selectivity to odour compounds or ease of calibration, especially in the context of relating and validating the sensor response signals to the presence of odours. It will not be applicable to the measurement of hedonic values such as measurement of odour concentration, which is the task of other work groups, such as CEN/TC264/WG2, which aims to revise the EN13725 standard for determination of odour concentration by dynamic olfactometry [106].

Stability upon temperature and humidity changes, as well as sensor response drift in time are still fundamental problems connected with the sensors comprising the electronic nose instruments. Bioelectronic noses are met with optimism, which are a subgroup of the electronic noses. Their specificity and high sensitivity can lead to the elaboration of commercial versions. Numerous literature reports concerning fundamentals and potentialities of electronic and bioelectronic noses justify the statement that development of the methods of odours analysis is connected with the utilization of such type of devices.

## Figures and Tables

**Table 1:** Olfactory thresholds for the chemical compounds detected in the vicinity of municipal processing plants [26,27,28] containing particular osmophore groups.

Osmophoric Group	Example Compound	Odour Threshold Value	Reference
Alcohols	methanol	33 ppm	[26]
	ethanol	55 ppm	[27]
	propanol	6 ppm	[27]
Aldehydes	formaldehyde	0.83 ppm	[28]
	acetaldehyde	0.21 ppm	[16]
	n-butylaldehyde	0.67 ppb	[26]
Ketones	acetone	13.5 ppm	[20]
	n-butanone	50 ppm	[26]
	3-pentanone	70 ppm	[26]
Acids	aceticacid	363 ppb	[27]
	propanoicacid	5.7 ppb	[26]
	butyricacid	1 ppb	[16]
Esters	ethyl acetate	3.9 ppm	[28]
	butylacetate	0.39 ppm	[28]
	methylmethacrylate	0.21 ppm	[16]
Amines/amides	methylamine	35 ppb	[26]
	trimethylamine	0.21 ppb	[16]
	dimethylformamide	2.2 ppm	[28]
Sulphur compounds	dimethylsulphide	5.89 ppb	[27]
	dimethyldisulphide	0.16 ppb	[26]
	methyl mercaptane	2.1 ppb	[16]

**Table 2:** Commercially available chemical sensors intended for measurement of compounds from volatile organic compounds group characterized by the limit of detection lower than 1 ppm *v*/*v*.

Manufacturer/Model	Sensor Type ^1^	Measuring Range	Response Time	Detected Compound
Winsen, ME2-C2H5OH-Ф16	EC	0–1 mg/dm^3^	<20 s	ethanol
Winsen, ME2-CH2O-Ф16	EC	0–10 ppm	<60 s	formaldehyde
Environmental Sensors CO, Z-300	EC	0–30 ppm	<60 s	formladehyde
3ETO CiTiceL	EC	0.1–20 ppm	<140 s	ethylene oxide
Membrapor, ETO/M-10	EC	0.05–10 ppm	<140 s	ethylene oxide
Uni-tec SRL, SENS-IT	MOS	0.1–30 ppb	nd	benzene
Uni-tec SRL, SENS 3000	MOS	0–400 ppb	<3 s	methane
UST, Triplesensor	MOS	0.1–100 ppm	<100 s	benzene
Alphasense, PID-A12	PID	0.001–50 ppm	<3 s	VOCs with ionisation potentials < 10.6 eV (isobutylene calibration)
Alphasense, PID-AH	PID	0.001–50 ppm	<3 s	VOCs with ionisation potentials < 10.6 eV (isobutylene calibration)
ION Science, PPB MiniPID 2	PID	0.001–40 ppm	<3 s	VOCs (isobutylene calibration)
piD-TECH eVx, Blue 045-014	PID	0.0005–1 ppm	<4 s	VOCs (isobutylene calibration)
piD-TECH plus, 043-235	PID	0.005–20 ppm	<5 s	VOCs (isobutylene calibration)

^1^ EC—Electrochemical sensor, MOS—Metal Oxide Semiconductor, PID—Photoionization sensor.

**Table 3:** Examples of application of electronic nose instruments for evaluation of air odour quality in a vicinity of municipal processing plants.

Application	Type	Level of Advancement	Results Expressed in Odour Concentration Units	Comparision with Olfactometry	Sensors	Data Processing	Reference
**NETWORK**							
Indoor/outdoor air quality	prototype	Advanced in-situ experiments	No	No	MOS	Neural Processing Blocks (NPB)	[43]
Odour dispersions modelling	commercial	In-situ implementation	Yes	Yes	Odotech’s system, OdoWatch	mapping	[44]
Pulp and paper industry	prototype	Calibration of electronic noses network	No	No	MOS	ANN	[77]
Odour monitoring	prototype	In-situ implementation	No	Yes	MOS	cluster analysis, mapping	[78]
Assessment of odour annoyance near a compost facility.	prototype	Advanced in-situ experiments	Yes	Yes	MOS	DFA, PLS	[79]
Monitoring of odours from a composting plant	commercial	Advanced in-situ experiments	Yes	Yes	EOS, Sacmi Group, Imola, Italy (MOS)	kNN	[80]
Monitoring odour emissions from an oil & gas plant	commercial	Advanced experiments in model condition	Yes	Yes	EOS Ambiente, Sacmi Group, Imola, Italy (MOS)	not provided	[81]
**PORTABLE**							
Landfills odour monitoring	prototype	Improvement of electronic nose in field studies	No	No	MOS	DA, PCA, MLR, PCR, PLS	[82]
Outdoor air quality	prototype	Basic in-situ experiments	No	No	MOS, EC	mapping	[83]
Odour measurement around: compost facilities, printing houses, paint shops, wastewater treatment plants, rendering plants, settling ponds of sugar factories	prototype	Basic in-situ experiments	No	No	MOS	DA, PCA	[84]
Unpleasant and potentially harmful odours in urban areas, likely coming from residential waste containers	prototype	Basic in-situ experiments	No	No	MOS	mapping	[85]
Farm odour	prototype	Advanced in-situ experiments	Yes	Yes	MOS	ANN	[86]
Asphalt odour patterns in hot mix asphalt production	commercial	Basic experiments in model condition	No	No	Cyranose 320, Smiths Detection Inc., Edgewood, MD, USA (CP)	Polar plots, PCA	[87]
**ROBOTS & DRONES**							
Air Quality	prototype	Basic experiments in model condition	No	No	MOS	mapping	[88]
Localizing gas emission sources on landfill sites	prototype	Advanced experiments in model condition	No	No	MOS	Polar plots & mapping	[62]
Air Quality	prototype	Basic experiments in model condition	No	No	MOS	mapping	[89]

ANN—Artificial Neural Network; CP—Conducting Polymer; DA—Discriminant Analysis; DFA—Discriminant Function Analysis; kNN—k-Nearest Neighbours; MOS—Metal Oxide Semiconductor; MLR—Multiple Linear Regression; PCA—Principal Component Analysis; PCR—Principal Component Regression; PLS—Partial Least Squares.
